# Antitumor activity of motesanib alone and in combination with cisplatin or docetaxel in multiple human non–small-cell lung cancer xenograft models

**DOI:** 10.1186/1476-4598-11-70

**Published:** 2012-09-19

**Authors:** Angela Coxon, Beth Ziegler, Stephen Kaufman, Man Xu, Hongyu Wang, Dawn Weishuhn, Joanna Schmidt, Heather Sweet, Charlie Starnes, Douglas Saffran, Anthony Polverino

**Affiliations:** 1 Department of Oncology Research, Amgen Inc, One Amgen Centre Drive, Thousand Oaks, CA 91320, USA; 2 Department of Pathology, Amgen Inc, One Amgen Centre Drive, Thousand Oaks, CA, 91320, USA; 3 Department of Oncology Research, Amgen Inc, 360 Binney Street, Cambridge, MA, 02142, USA; 4 Department of Protein Sciences, Amgen British Columbia, 7990 Enterprise St, Burnaby, BC, V5A 1V7, Canada; 5 Department of Oncology Research, Amgen Inc, 1201 Amgen Court West, Seattle, WA, 98119, USA

**Keywords:** Motesanib, Cisplatin, Docetaxel, VEGF receptor, NSCLC, Angiogenesis

## Abstract

**Background:**

Non–small-cell lung cancer (NSCLC) is categorized into various histologic subtypes that play an important role in prognosis and treatment outcome. We investigated the antitumor activity of motesanib, a selective antagonist of vascular endothelial growth factor receptors (VEGFR) 1, 2, and 3, platelet-derived growth factor receptor, and Kit, alone and combined with chemotherapy in five human NSCLC xenograft models (A549, Calu-6, NCI-H358, NCI-H1299, and NCI-H1650) containing diverse genetic mutations.

**Results:**

Motesanib as a single agent dose-dependently inhibited tumor xenograft growth compared with vehicle in all five of the models (*P* < 0.05). When combined with cisplatin, motesanib significantly inhibited the growth of Calu-6, NCI-H358, and NCI-H1650 tumor xenografts compared with either single agent alone (*P* < 0.05). Similarly, the combination of motesanib plus docetaxel significantly inhibited the growth of A549 and Calu-6 tumor xenografts compared with either single agent alone (*P* < 0.05). In NCI-H358 and NCI-H1650 xenografts, motesanib with and without cisplatin significantly decreased tumor blood vessel area (*P* < 0.05 vs vehicle) as assessed by anti-CD31 staining. Motesanib alone or in combination with chemotherapy had no effect on tumor cell proliferation in vitro.

**Conclusions:**

These data demonstrate that motesanib had antitumor activity against five different human NSCLC xenograft models containing diverse genetic mutations, and that it had enhanced activity when combined with cisplatin or docetaxel. These effects appeared to be mediated primarily by antiangiogenic mechanisms.

## Background

Lung cancer is the primary cause of cancer death and the second most frequent cause of new cancer cases in the United States [1]. The majority of these cases are non–small-cell lung cancer (NSCLC) [2], which comprises nonsquamous carcinoma (including adenocarcinoma, large cell carcinoma, and other cell types) and squamous cell carcinoma [3]. Survival among patients with advanced NSCLC is poor with currently recommended doublet chemotherapy regimens [3,4]. Targeted therapies, and particularly those that inhibit angiogenesis, are being actively explored as alternative treatment options [5].

Vascular endothelial growth factor (VEGF), a proangiogenic cytokine, is frequently overexpressed in NSCLC tumors, and its overexpression is associated with increased microvessel density [6-8]. Furthermore, high VEGF expression has been associated with nodal metastasis, poor prognosis, and reduced survival in NSCLC [6,7,9]. Bevacizumab, a monoclonal antibody targeting VEGF-A, has been shown to improve overall survival when administered with carboplatin/paclitaxel [10] and to prolong progression-free survival in combination with gemcitabine/cisplatin [11] in patients with nonsquamous histology.

There is increasing evidence that NSCLC histologic subtype is useful for predicting patient outcome and clinical benefit from treatment with cytotoxic and argeted cancer therapies [12-14]. In a recent retrospective analysis of the phase 3 E4599 study of bevacizumab combined with carboplatin/paclitaxel in nonsquamous NSCLC, median overall survival was 14.2 months in the bevacizumab arm compared with 10.3 months in the control arm among patients with adenocarcinoma (hazard ratio [95% CI], 0.69 [0.58–0.83]). In contrast, among patients with large cell carcinoma, median overall survival was 10.0 months in the bevacizumab arm and 8.7 months in the control arm (hazard ratio [95% CI], 1.15 [0.60–2.24]) [15]. Prognosis and response to targeted treatment also appear to be influenced by a number of characteristic NSCLC driver mutations that are thought to be responsible for the initiation and maintenance of the malignancy. The most common are somatic mutations in the *KRAS*, *EGFR*, and *ALK* genes but mutations in other genes, including *BRAF*, occur as well [16]. It is well established that *EGFR* mutations can significantly predict patient outcome and response to the epidermal growth factor receptor (EGFR) inhibitor gefitinib in Asian patients with advanced NSCLC [17-20]. The potentially predictive value of other NSCLC driver mutations is still under investigation [21,22]. The emerging significance of both histologic subtype and presence of mutations in NSCLC suggests that testing of novel targeted therapies in preclinical models of varying histologies and genetic backgrounds may be a critical step in the evaluation process.

Motesanib is a small-molecule antagonist of VEGF receptors (VEGFR) 1, 2, and 3; platelet-derived growth factor receptor (PDGFR); and Kit [23]. In tumor xenograft models, including models of thyroid cancer and breast cancer, oral administration of motesanib, alone or in combination with chemotherapy resulted in tumor regression and inhibition of angiogenesis [23-26]. In phase 1 and phase 2 studies in advanced solid tumors including advanced nonsquamous NSCLC, motesanib as a monotherapy and combined with chemotherapy has shown evidence of antitumor activity [27-31].

The objective of the present study was to investigate the antitumor activity of motesanib as monotherapy and in combination with cisplatin or docetaxel in five human NSCLC xenograft models of varying histologic subtypes and genetic backgrounds, with the hypothesis that combined treatment would improve antitumor activity over that of single-agent treatment. Motesanib had antiangiogenic and antitumor activity against all five human NSCLC models and had enhanced antitumor activity when combined with cisplatin or docetaxel chemotherapy.

## Results

### Expression of VEGFR2 and effect of motesanib on NSCLC tumor cell proliferation

**Figure 1:**
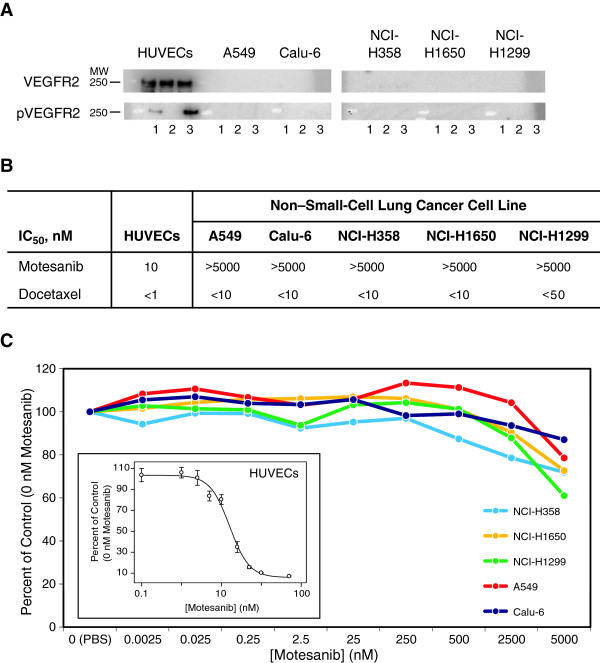
**Expression of VEGFR2 and effect of motesanib on NSCLC tumor cell proliferation in vitro.** (**A**) Relative expression levels of total and phosphorylated human VEGFR2 protein by HUVECs, A549, Calu-6, NCI-H358, NCI-H1650, and NCI-H1299 cells in full-serum media (lane 1), serum-free conditions (lane 2), or serum-free conditions with 50 ng/mL recombinant human VEGF (lane 3). (**B**) In vitro proliferation of HUVECs, A549, Calu-6, NCI-H358, NCI-H1650, and NCI-H1299 cells in full-serum conditions or (HUVECs only) in serum-free conditions with 50 ng/mL recombinant human VEGF after addition of motesanib (at concentrations of 0.0025 to 5000 nM) or docetaxel (at concentrations of 0.001 to 5000 nM). (**C**) IC_50_ curves for in vitro proliferation of each of the cell lines shown in panel **B**. IC_50_ = Half-maximal inhibitory concentration.

Western blot analysis failed to detect expression of total or phosphorylated VEGFR2, one of the molecular targets of motesanib, in A549, Calu-6, NCI-H358, NCI-H1299, or NCI-H1650 cells in full-serum media, following serum starvation, or after treatment with recombinant human VEGF (50 ng/mL) for 5 minutes. In contrast, cultured human umbilical vein endothelial cells (HUVECs) expressed total VEGFR2 in all three culture conditions and phosphorylated VEGFR2 in full-serum conditions which was further increased after stimulation with recombinant human VEGF (Figure 1A). Microarray analyses showed that A549, Calu-6, NCI-H1299, and NCI-H1650 tumors expressed similar levels of VEGF mRNA; and that A549, Calu-6, NCI-H1299, and NCI-H1650 tumors expressed VEGFR1, 2, and 3 mRNA (data not shown). A549 was the only line to express PDGFRα, and none of the cell lines expressed PDGFRβ or Kit (data not shown; NCI-H358 cells were not tested). In vitro assays demonstrated that 5 μM motesanib had no effect on the proliferation of A549, Calu-6, NCI-H358, NCI-H1299, and NCI-H1650 tumor cells but inhibited the proliferation of endothelial cells (IC_50_, 10 nM) (Figure 1B). In the same experiment, the cytotoxic chemotherapy agent docetaxel inhibited proliferation of each of the cultured cell lines, including HUVECs with IC_50_ values in the low nanomolar range (Figure 1B). These data do not support a direct antitumor effect of motesanib on NSCLC cells.

### Effect of single-agent motesanib on human NSCLC tumor growth

**Figure 2:**
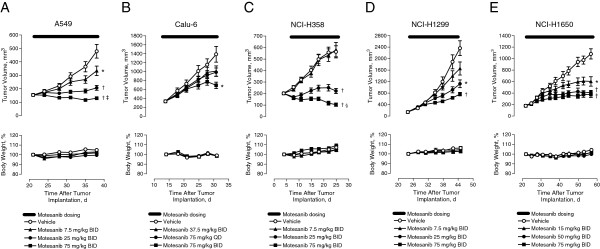
**Effect of single-agent motesanib on various NSCLC xenograft tumor models.** Tumor volume (top) and body weight (bottom) over time in response to single-agent motesanib. (**A**) A549 tumors treated with vehicle or motesanib 7.5 mg/kg BID, 25 mg/kg BID, or 75 mg/kg BID for 17 days. ^‡^P < 0.01 regression; day 21 vs day 38. (**B**) Calu-6 tumors treated with vehicle or motesanib 37.5 mg/kg BID, 75 mg/kg QD, or 75 mg/kg BID for 17 days. (**C**) NCI-H358 tumors treated with vehicle or motesanib 7.5 mg/kg BID, 25 mg/kg BID, or 75 mg/kg BID for 21 days. ^§^P < 0.0001 regression; day 4 vs day 25. (**D**) NCI-H1299 tumors treated with vehicle or motesanib 7.5 mg/kg BID, 25 mg/kg BID, or 75 mg/kg BID for 21 days. (**E**) NCI-H1650 tumors treated with vehicle or motesanib 15 mg/kg BID, 50 mg/kg BID, or 75 mg/kg BID for 35 days. Data are expressed as mean ± SE. For all panels, *P < 0.05 vs vehicle, ^†^P < 0.0001 vs vehicle.

The effect of motesanib on NSCLC tumor growth in vivo was assessed using A549, Calu-6, NCI-H358, NCI-H1299, and NCI-H1650 subcutaneous tumor xenografts. Treatment with motesanib significantly inhibited growth in each of these models. In the A549, NCI-H1299, and NCI-H1650 xenograft models, motesanib demonstrated a dose-dependent effect on tumor growth. In mice with established A549 tumors, all three doses of motesanib tested (7.5, 25, and 75 mg/kg BID) significantly inhibited tumor growth (45%, 84%, and 107%, respectively), compared with vehicle (Figure 2A). In animals bearing Calu-6 tumors, a significant inhibitory effect of motesanib on tumor growth (66% inhibition) was only seen at the highest dose tested of 75 mg/kg twice daily (BID) for 17 days compared with vehicle (Figure 2B). In the NCI-H358 xenograft model, significant inhibition of tumor growth (94% and 127%, respectively) compared with vehicle was seen at the two highest motesanib dose levels (25- and 75-mg/kg BID; Figure 2C). In the NCI-H1299 xenograft model, significant inhibition of tumor growth (56% and 72%, respectively) compared with vehicle was observed at the two highest motesanib dose levels (25- and 75-mg/kg BID; Figure 2D). In mice bearing NCI-H1650 xenografts, motesanib at all three administered doses (15, 50, or 75 mg/kg BID) significantly inhibited tumor growth (45%, 67%, and 78%, respectively) compared with vehicle (Figure 2E). In all treatment groups across the various models, motesanib was well tolerated. Body weights remained stable over the treatment periods and were similar to those of vehicle-treated control animals (Figure 2). Similar effects of VEGF inhibition on tumor growth were observed in a *KRAS*-driven genetically engineered mouse model of lung adenocarcinoma (see Additional file 1).

Table 1 summarizes the mutational status of cells from the various tumor xenografts along with representative antitumor activity of motesanib in each model. DNA sequencing confirmed the presence of *KRAS* mutations, one of the most common driver mutations in lung adenocarcinoma, in three of the xenograft models and mutations in *BRAF* and *NRAS*, two less frequently occurring mutations associated with NSCLC [32], in two of the models. The functional significance of the *BRAF* mutation (heterozygous deletion of exon 2) is unknown. Notably, in all five NSCLC xenografts, motesanib monotherapy administered at 75 mg/kg BID inhibited tumor growth by at least 66%, suggesting that motesanib has broad antitumor activity independent of the mutational characteristics of the NSCLC cells.

**Table 1:** Mutational status of NSCLC xenograft tumor models and associated motesanib antitumor activity

**Xenograft tumor model**	**Tumor volume at initiation of therapy (mm**^**3**^**)**	**Motesanib monotherapy dose (mg/kg BID)**	% Tumor growth inhibition*	**Confirmed mutations**^**†**^
A549	153	75	107	*KRAS, STK11*
Calu-6	336	75	66	*KRAS, TP53*
NCI-H358	202	75	127	*KRAS, TP53*^§^
NCI-H1299	141	75	72	*NRAS, TP53*^§^
NCI-H1650	179	75^‡^	78	*EGFR, BRAF, TP53*^*¶*^

BID, twice daily.

*Data from a single experiment.

^†^By DNA sequencing (see *Methods, Cell Lines and Reagents*).

^‡^Based on once daily administration.

^§^NCI-H358 and NCI-H1299 cells are *TP53* null per previously published literature [52,53].

^*¶*^The functional significance of the *BRAF* mutation (heterozygous deletion of exon 2) is unknown. NCI-H1650 cells have been reported to carry mutations in *CDKN2A*, *EGFR*, and *TP53*[50,51].

### Effect of motesanib in combination with cisplatin on human NSCLC tumor growth

**Figure 3:**
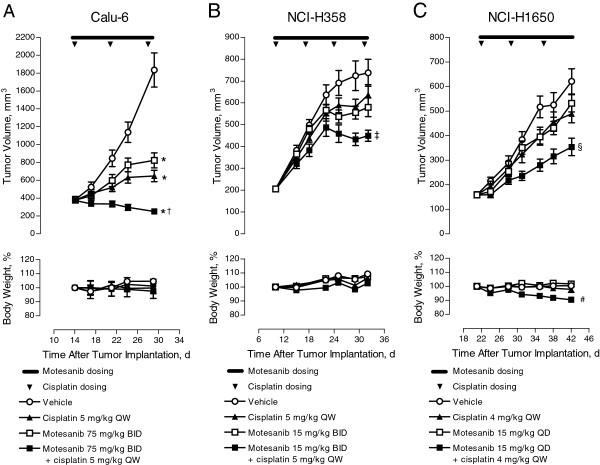
**Effect of motesanib in combination with cisplatin on various NSCLC xenograft tumor models.** Tumor volume (top) and body weight (bottom) over time in response to motesanib plus cisplatin. (**A**) Calu-6 tumors treated with vehicle, motesanib 75 mg/kg BID, cisplatin 5 mg/kg QW, or motesanib plus cisplatin at the same dose and schedule for 14 days. *P < 0.0001 for all groups vs vehicle; ^†^P *≤* 0.05 for combination vs either single agent alone. (**B**) NCI-H358 tumors treated with vehicle, motesanib 15 mg/kg BID, cisplatin 5 mg/kg QW, or motesanib plus cisplatin at the same dose and schedule for 22 days. ^‡^P ≤ 0.001 for combination vs either single agent alone; P < 0.0001 for combination vs vehicle. (**C**) NCI-H1650 tumors treated with vehicle, motesanib 15 mg/kg QD, cisplatin 4 mg/kg QW, or motesanib plus cisplatin at the same dose and schedule for 21 days. ^§^P = 0.001 for combination vs either single agent alone; P < 0.0001 for combination vs vehicle. ^#^P < 0.0001 for combination vs vehicle, motesanib, or cisplatin alone. Data are expressed as mean ± SE.

The antitumor activity of motesanib or cytotoxic chemotherapy alone in Calu-6, NCI-H358, and NCI-H1650 xenograft models was enhanced by the combined administration of both agents. In some of these experiments, suboptimal doses of motesanib (based on previous dose–response data) were used to allow for the observation of additive activity. In animals bearing established Calu-6 tumors, motesanib (75 mg/kg BID) or cisplatin (5 mg/kg QW), a standard-of-care chemotherapy agent in NSCLC, significantly inhibited tumor growth compared with vehicle. However, when both agents were combined at the same dose and schedule as in the monotherapy experiments, tumor growth inhibition was significantly greater than that observed with either single agent alone (Figure 3A). Similarly, in mice bearing NCI-H358 tumors, combination treatment with motesanib (15 mg/kg BID) and cisplatin (5 mg/kg QW) resulted in significantly greater tumor growth inhibition than that achieved with either monotherapy (Figure 3B). In these two models, both treatment modalities (monotherapy and the combination of motesanib plus cisplatin) appeared tolerable as there were no significant changes in the animals’ body weight over the course of the experiments (Figures 3A and 3B).

Similar cooperative activity was observed in mice bearing established NCI-H1650 tumors. As seen with the other two xenograft models, combining both motesanib (15 mg/kg QD) and cisplatin (4 mg/kg QW) resulted in significantly greater tumor growth inhibition than that measured with either agent alone (Figure 3C). However, animals that received combination therapy showed a significant reduction from baseline (approximately 10%) in body weight (*P* < 0.0001 vs vehicle, motesanib alone, and cisplatin alone; Figure 3C). The differential effects of motesanib and cisplatin on body weights in the NCI-H358 and NCI-H1650 models suggests that the mechanism of body weight loss observed in the NCI-H1650 model is not a general phenomenon related to the combination of motesanib and cisplatin. Efforts to understand this differential activity are being explored.

### Effect of motesanib in combination with docetaxel on human NSCLC tumor growth

**Figure 4:**
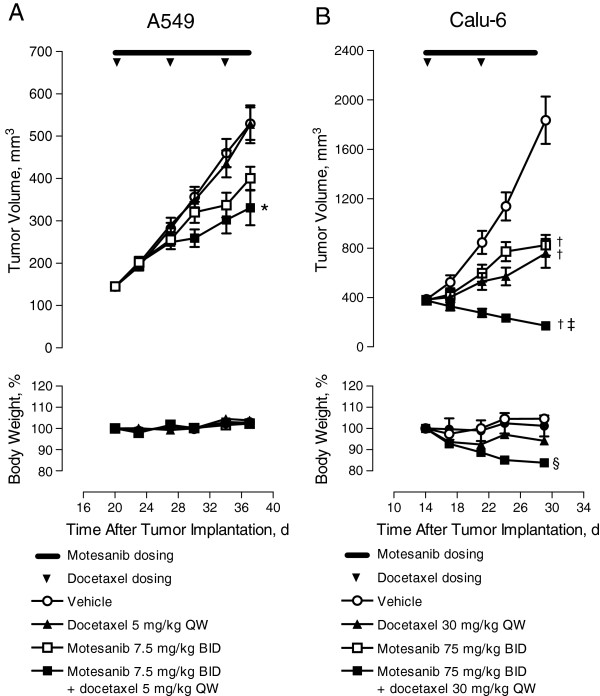
**Effect of motesanib in combination with docetaxel on various NSCLC xenograft tumor models.** Tumor volume (top) and body weight (bottom) over time in response to motesanib plus docetaxel. (**A**) A549 tumors treated with vehicle motesanib 7.5 mg/kg BID, docetaxel 5 mg/kg QW, or motesanib plus docetaxel at the same dose and schedule for 14 days. *P ≤ 0.02 for combination vs either single agent alone. (**B**) Calu-6 tumors treated with vehicle, motesanib 75 mg/kg BID, docetaxel 30 mg/kg QW, or motesanib plus docetaxel at the same dose and schedule for 17 days. ^‡^P < 0.005 for combination vs either single agent alone. ^†^P < 0.0001 vs vehicle; ^§^P < 0.0001 for combination vs vehicle or either single agent. Data are expressed as mean ± SE.

Experiments parallel to those described above were performed for motesanib and docetaxel, another standard-of-care chemotherapy in the treatment of NSCLC, using the A549 and Calu-6 tumor xenograft models. To allow for the observation of additive activity, suboptimal doses of motesanib were used in some models based on previous dose–response data. As seen with cisplatin, the antitumor activity of the combined treatment modality was greater than that for either agent alone. In mice bearing A549 tumors, treatment with motesanib (7.5 mg/kg BID) combined with docetaxel (5 mg/kg QW) resulted in significantly greater inhibition of tumor growth than either agent alone (Figure 4A). In this experiment, both monotherapy and combination regimens had no adverse effect on the body weight of the animals (Figure 4A). Additive antitumor efficacy was also observed in the Calu-6 xenograft model. In mice bearing Calu-6 tumors, motesanib (75 mg/kg BID) or docetaxel (30 mg/kg QW) alone significantly inhibited tumor growth, and that effect was even greater when both agents were combined (Figure 4B). However, the combination of motesanib and docetaxel in the Calu-6 model was associated with a significant decrease from baseline in mean body weight (approximately 15%; Figure 4B).

**Figure 5:**
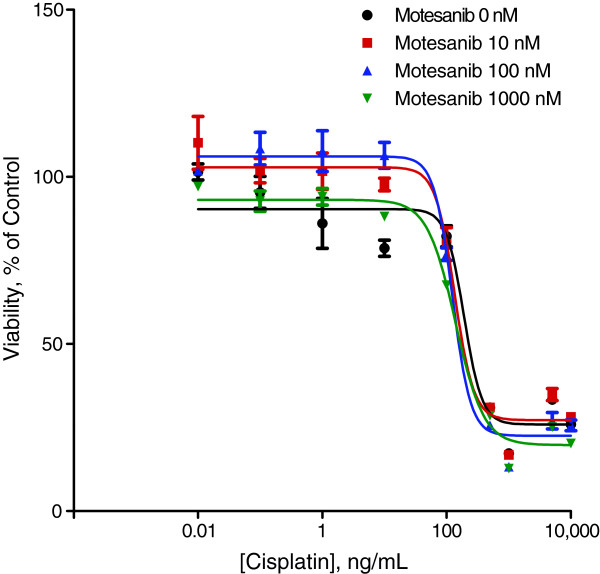
**Effect of motesanib plus cisplatin treatment on cell proliferation in vitro.** Calu-6 cells plated at 3000 cells/well in 96 well plates were treated with motesanib (0, 10, 100, or 1000 nM) plus serial dilutions of cisplatin (0.01 to 10,000 ng/mL) for 72 hours. Cell viability was assessed using the ATPlite™ 1-step luminescence assay (PerkinElmer, Waltham, MA) and is expressed as a percentage of control (ie, 0 ng/mL cisplatin). Identical experiments were performed using A549, NCI-H358, NCI-H1299, and NCI-H1650 cells (not shown).

To better understand the enhanced antitumor efficacy in vivo, we performed a separate set of experiments to test whether treatment with motesanib plus chemotherapy had a direct effect on the proliferation of the different NSCLC cell lines in vitro. There was no difference in cell viability between cisplatin or docetaxel single-agent and motesanib/chemotherapy combination treatment. Results from a representative experiment with cisplatin and motesanib using Calu-6 cells are shown in Figure 5.

We also investigated the possibility that the measured treatment effect of motesanib plus chemotherapy on the various tumor xenograft models was the result of changes in the plasma exposure of the respective agents. No consistent variations in the pharmacokinetics of motesanib or either of the chemotherapy agents when administered in combination were noted in the NSCLC models tested here (data not shown). This is in line with earlier experiments using xenograft models of other tumor types, reporting no significant changes in the pharmacokinetics of motesanib and docetaxel when administered alone or in combination [24].

### Histologic analysis of NSCLC tumor xenografts treated with motesanib plus cisplatin

**Figure 6:**
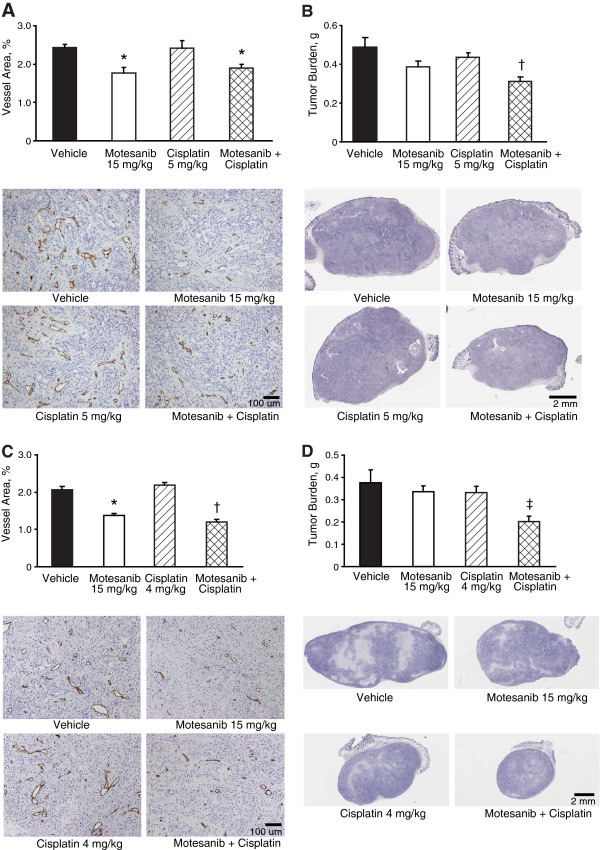
**Histomorphometric analyses of viable tumor burden and tumor blood vessel area in representative NSCLC xenografts treated with motesanib and/or cisplatin.** Representative images and quantification of tumor blood vessel area (**A**) and viable tumor burden (**B**) from NCI-H358 xenografts treated with vehicle, motesanib 15 mg/kg BID, cisplatin 5 mg/kg QW, or motesanib plus cisplatin at the same dose and schedule. *P < 0.05 vs vehicle; ^†^P < 0.05 vs vehicle and cisplatin monotherapy. Representative images and quantification of tumor blood vessel area (**C**) and viable tumor burden (**D**) from NCI-H1650 xenografts treated with vehicle, motesanib 15 mg/kg QD, cisplatin 4 mg/kg QW, or motesanib plus cisplatin at the same dose and schedule. ^*^P < 0.0001 vs vehicle; ^†^P < 0.0001 vs vehicle and cisplatin monotherapy; ^‡^P = 0.0039 vs vehicle. Data are expressed as mean ± SE (panel **A**, n = 6; panel **B**, n = 10; panel **C**, n = 10; panel **D**, n = 10). Bar, 100 μm (panels **A** and **C**) or 2 mm (panels **B** and **D**).

Tumor morphology was examined to explore the potential mechanisms of action of motesanib combined with cisplatin. Immunohistochemical staining of NCI-H358 and NCI-H1650 tumors at the end of the experiments (day 33 and day 43, respectively) demonstrated that the vessel area percentage was significantly decreased after treatment with the suboptimal dose of motesanib (15 mg/kg) alone or in combination with cisplatin at 5 mg/kg (NCI-H358; Figure 6A) or 4 mg/kg (NCI-H1650; Figure 6C), compared with vehicle or cisplatin monotherapy. In addition, tumor burden (tumor viable fraction × tumor weight) was significantly decreased in both the NCI-H358 and the NCI-H1650 xenograft models when motesanib plus cisplatin was administered in combination, compared with either vehicle or cisplatin monotherapy (Figures 6B and 6D). In contrast, at the doses tested tumor burden was not decreased in either xenograft model when motesanib or cisplatin were given as monotherapy, presumably because both agents were used at suboptimal doses.

## Discussion

Recently, there has been increased recognition of histologic subtype as a potential predictor of efficacy with first-line treatment of advanced NSCLC [12,13]. A retrospective analysis of the pivotal E4599 trial of first-line bevacizumab combined with carboplatin/paclitaxel in nonsquamous NSCLC showed that patients with adenocarcinoma (the most frequent histologic subtype) who received bevacizumab combined with carboplatin/paclitaxel had favorable overall survival compared with patients who received carboplatin/paclitaxel alone [15]. Although the number of enrolled patients with other histologic subtypes was small, clinical benefits among these patients appeared more limited than among those with adenocarcinoma. Current multidisciplinary recommendations for changes in NSCLC classification [33] support the potential value of histologic subtype in influencing treatment decisions in NSCLC. In addition to histology, research has revealed the importance of driver mutations in NSCLC, which are particularly prevalent among adenocarcinomas, presenting at different incidence rates in specific patient groups. Preclinical and clinical evidence has shown that these mutations may affect response to targeted therapy [16,22], suggesting that a further division of NSCLC into clinically relevant mutational subgroups may allow for a more tailored treatment approach. Targeted treatments that may be effective regardless of mutational status may be even more desirable.

Hence, it appears critical that preclinical studies assessing investigational agents should be designed to test activity in models that represent more than one NSCLC subtype. The antitumor activity of a number of VEGFR inhibitors either as single agents or combined with chemotherapy has been demonstrated in a variety of NSCLC xenograft models [34-38]. However, few of these studies have assessed activity in more than one or two different models. The present study aimed to assess the antitumor activity of motesanib as a single agent or combined with chemotherapy in human NSCLC xenograft models with varying genetic backgrounds and histology [36,39-41]. When administered alone, motesanib inhibited the growth of all five NSCLC xenografts, A549, Calu-6, NCI-H358, NCI-H1299, and NCI-H1650, in a dose-dependent manner. Calu-6 tumors were relatively resistant to treatment compared with the other cell lines: only the highest motesanib dose administered (75 mg/kg BID) resulted in xenograft growth inhibition (66%). Decreased responsiveness to single-agent VEGFR inhibitors, including motesanib, and epidermal growth factor receptor (EGFR) inhibitors in the Calu-6 model (compared with other tumor xenograft models) have been described previously [34,35,42]. The reasons for this differential responsiveness are not immediately evident, but based on the above studies, it is likely rooted in causes other than variations in experimental design, because it has been seen across independent studies and with various therapies focusing on different molecular targets. In all models, tumor xenograft growth inhibition increased when motesanib was combined with QW cisplatin or docetaxel, agents that are components of standard two-drug chemotherapies for NSCLC treatment [4], compared with either single-agent treatment. The results from the experiment with the Calu-6 model are particularly noteworthy because of its relative resistance to treatment with angiogenesis inhibitors and EGFR-targeted agents alone. One possible explanation for the improved antitumor activity of the combination treatments is that angiogenesis inhibitors may modulate the tumor vasculature, resulting in enhanced delivery of chemotherapy to target cells [43,44], but we have not directly addressed this issue in the current study. Mutational status of the cells appeared to have had no influence on the antitumor activity of motesanib treatment. Regardless of whether cell lines had common driver mutations (ie, *KRAS*) or less frequently occurring mutations (ie, *BRAF*, *NRAS*) or a combination of mutated genes, treatment with motesanib as monotherapy or in combination with chemotherapy resulted in tumor growth inhibition, albeit at different doses depending on the model. Targeted treatments that may be effective regardless of mutational status of the patient may be particularly desirable, particularly for agents targeting the stroma, where the mutational spectrum is not expected to be equivalent.

The antitumor activity that motesanib exhibited against the NSCLC xenograft models chosen for this study was mediated, at least in part, by an antiangiogenic mechanism rather than a direct effect on the tumor cells themselves. Unlike human medullary cancer cells that express VEGFR2 [26], VEGFR2 could not be detected in any of the cell lines and phosphorylated VEGFR2 could not be detected following exogenous administration of VEGF. Motesanib did not inhibit the proliferation of cells from any of the cell lines in vitro. The lack of inhibition of proliferation also suggests that other motesanib targets (eg, PDGFR and Kit) are not important in this context. It should be noted that some studies have reported that sorafenib and vandetanib (both of which inhibit VEGFR signalling) can attenuate the proliferation of lung cancer cells in vitro [45-47]. However, both of these agents also inhibit EGFR signalling and, consequently, it is not possible to ascertain whether the observed effects were due to inhibition of VEGFR signalling, EGFR signalling, or both. Treatment with single-agent motesanib or motesanib plus cisplatin showed significant reductions in tumor blood vessel area compared with vehicle in NCI-H358 and NCI-H1650 xenografts. These results are consistent with those from previous studies reporting that motesanib alone or combined with chemotherapy had antitumor activity in xenograft models of breast, thyroid, and colorectal cancer, which was also associated with a significant decrease in tumor blood vessel area [23-26]. Overall, our data support a predominant role for antiangiogenesis in inhibition of tumor growth by motesanib.

## Conclusions

Our data show that motesanib has antiangiogenic and antitumor activity in all five tested NSCLC subcutaneous xenograft models of varying histologic subtypes and genetic backgrounds. When combined with cisplatin or docetaxel, the antitumor activity of motesanib was significantly greater than single-agent treatment in each of the four xenograft models in which combination treatments were tested. Investigation of their activity in xenograft models with a variety of histologic subtypes is a valuable and appropriate strategy for preclinical assessment of anticancer agents in NSCLC.

## Methods

### Cell lines and reagents

Non–small-cell lung cancer cell lines including A549 carcinoma, Calu-6 anaplastic carcinoma, NCI-H358 bronchioalveolar carcinoma, NCI-H1299 lung carcinoma, and NCI-H1650 bronchioalveolar adenocarcinoma cells were originally obtained from the American Type Culture Collection (Manassas, VA) between 2001 and 2008. A549, Calu-6, NCI-H358, and NCI-H1299 cell lines were tested and authenticated, at the time of the experiments, by DNA sequencing of the following genes, which confirmed the presence of specific mutations equivalent to those previously described for these cells [48,49]: *KRAS*, *NRAS*, *EGFR*, *BRAF*, *P53*, *PTEN*, *cMET*, *PIK3CA*, and *STK11* (see also Table 1). NCI-H1650 cells have been reported to carry mutations in *CDKN2A*, *EGFR*, and *TP53*[50,51]. We did not sequence these genes in this cell line but did identify an additional mutation in *BRAF* (heterozygous deletion of exon 2). NCI-H358 and NCI-H1299 cells are *TP53* null per previously published literature [52,53]. Cells were maintained at 37°C in an atmosphere of 95% air and 5% CO_2_. A549 cells were cultured in F-12K nutrient medium with 10% fetal bovine serum (FBS) and 2 mM l-glutamine (Invitrogen Corp., Carlsbad, CA). Calu-6, NCI-H358, NCI-H1299, and NCI-H1650 cells were cultured in RPMI 1640 medium with 10% FBS and 2 mM l-glutamine; NCI-H1650 cultures were further supplemented with 1× nonessential amino acids. Human umbilical vein endothelial cells (HUVECs) were obtained from Lonza Walkersville Inc. (Walkersville, MD) and cultured in EGM-2 medium with EGM-2 SingleQuot supplement (Lonza Walkersville Inc.).

Docetaxel was obtained from Aventis Pharmaceuticals, Inc. (Bridgewater, NJ) and resuspended in PBS for in vitro cell assays. For in vivo assays, docetaxel was resuspended in the manufacturer-provided diluent and adjusted to the final concentration used before injection with phosphate-buffered saline (PBS). Cisplatin (1 mg/mL) was obtained from Bedford Laboratories (Bedford, OH) and APP Pharmaceuticals (Shaumburg, IL) and diluted in PBS for both in vitro and in vivo studies.

### In vitro cell proliferation assays

Cells (3,000/well) were seeded in 96-well plates using DMEM High-Glucose medium (Invitrogen Corp.) supplemented with 10% FBS and 2 mM l-glutamine (for A549, Calu-6, NCI-H358, NCI-H1299, and NCI-H1650 cells); or DMEM High-Glucose medium supplemented with 2 mM l-glutamine and 50 ng/mL of recombinant human VEGF (R&D Systems, Inc., Minneapolis, MN) (for HUVECs treated with motesanib). Cells were cultured overnight before being treated, in duplicate, with 10-point serial dilutions of single-agent motesanib (0.0025 to 5000 nM in medium containing 1% dimethyl sulfoxide [DMSO]) or docetaxel (0.001 to 5000 nM in PBS) for 72 hours at 37°C. Cell viability was mea-sured using an ATPlite™ 1-step luminescence assay (PerkinElmer, Waltham, MA) as described previously [24]. To assess the effect of motesanib plus chemotherapy combination treatment on in vitro proliferation, A549, Calu-6, NCI-H358, NCI-H1299, and NCI-H1650 cells were seeded as described above and then treated with motesanib (10, 100, and 1000 nM in medium containing 1% DMSO) plus serial dilutions of cisplatin or docetaxel in PBS for 72 hours at 37°C. Cell viability was determined using the ATPlite™ luminescence assay as described [24].

### In vitro tumor cell VEGFR2 phosphorylation

Phosphorylation of VEGFR2 in tumor cells and HUVECs was assessed as described [26]. Briefly, HUVECs, A549, Calu-6, NCI-H358, NCI-H1299, and NCI-H1650 cells were cultured in full-serum conditions, serum-starved conditions, and serum-starved conditions plus recombinant human VEGF at a final concentration of 50 ng/mL for 5 minutes before harvesting. Cells were lysed, and VEGFR2 protein was immunoprecipitated using an anti-human VEGFR2 polyclonal antibody (R&D Systems) and Protein A beads. Phosphorylated VEGFR2 protein was detected by Western blot using 4G10 horseradish peroxidase (HRP)–linked antiphosphotyrosine monoclonal antibody (Millipore, Billerica, MA). To detect total VEGFR2, the blot was stripped and reprobed with the polyclonal anti-VEGFR2 antibody (R&D Systems). Signals were detected with chemoluminescence using SuperSignal West Pico (Pierce Biotechnology Inc., Rockford, IL). Blot imaging was performed with a VersaDoc Imaging System Model 500 (Bio-Rad, Hercules, CA) and blot quantification with ImageQuant 5.2 software (Molecular Dynamics, Piscataway, NJ).

### Tumor xenograft models

Animals were obtained from the following sources: female CD1 nu/nu mice (Calu-6 models) from Charles River Laboratories (Raleigh, NC), female athymic nude mice (A549, NCI-H358, and NCI-H1299 models) from Harlan Sprague Dawley (Indianapolis, IN), and CB-17 severe combined immunodeficiency mice (NCI-H1650 models) from Charles River Laboratories (Montreal, QC, Canada). Procedures met the standards of the Amgen Animal Care and Use Committee. The facilities where experiments involving animals were conducted were approved by the Association for Assessment and Accreditation of Laboratory Animal Care.

On day 0, mice (approximately 5 to 9 weeks old) were injected subcutaneously on the right flank with either of the following: cultured Calu-6 (1 × 10^7^ cells in 200 μL of RPMI 1640 with 33% Matrigel [BD Biosciences, San Jose, CA]); A549 (5 x 10^6^ cells in 100 μL of F-12K with 50% Matrigel); NCI-H358 (5 × 10^6^ cells in 100 μL RPMI 1640 with 50% Matrigel); NCI-H1299 (5 × 10^6^ cells in 100 μL of RPMI 1640 with 50% Matrigel); or NCI-H1650 cells (5 × 10^6^ cells in 100 μL of RPMI 1640 with 50% Matrigel). After tumors became established, mice (9 to 10 per treatment group) received the following agents either alone or in combination as specified by the experimental protocols: vehicle (water, pH 2.5) or motesanib orally once daily (QD; 15 to 75 mg/kg) or twice daily (BID; 7.5 to 75 mg/kg); PBS or intraperitoneal cisplatin (4 or 5 mg/kg) once weekly (QW); or PBS or intraperitoneal docetaxel (5 or 30 mg/kg QW). For combination experiments, vehicle was added to the single agent dosing regimen at the appropriate route and schedule to match the combination group. Tumor dimensions were assessed twice weekly using an electronic digital caliper. For the Calu-6 tumor model, tumor volume was calculated as (length × width × height). For the A549, NCI-H358, NCI-H1299, and NCI-H1650 models, tumor volume was calculated as (width^2^ × length/2) where width was the smaller of two measurements and length was the larger of two measurements. All combination tumor studies were done in a blinded fashion. Body weight was recorded twice weekly as an index of toxicity.

### Tumor histology

The methods of tumor xenograft histologic examination have been described previously [24]. Briefly, tumors were removed at the end of experiments, weighed, bisected sagittally, and fixed in either zinc formalin (Anatech Ltd, Battle Creek, MI) or cold zinc-Tris fixative (BD Biosciences) and embedded in paraffin. Tumor sections fixed in zinc-Tris were immunostained for CD31 (vascular endothelium marker) using a monoclonal antibody (BD Biosciences, San Jose, CA) followed by 3,3’-diaminobenzidine (Dako Corp., Carpinteria, CA) as the chromogen. Tumor viability was assessed by hematoxylin staining. Tumor cross-sectional area and viable area were assessed by thresholding and automated pixel counting. The viable fraction was expressed as a percentage of total area. Estimated tumor burden was calculated as viable fraction × tumor weight. Scanned images of slides were analyzed using VisioMorph software v3.0.8.0 (Visiopharm, Horsholm, Denmark).

### Statistical analysis

The effects of single-agent or combination treatment with motesanib, cisplatin, or docetaxel on tumor growth and body weight were assessed by repeated-measures analysis of variance (RMANOVA) followed by Scheffé, Bonferroni/Dunn, or Dunnett post hoc testing using StatView software (version 5.0.1; SAS Institute, Inc., Cary, NC). For immunostaining, blood vessel area and viable tumor burden of tumors were compared by Student *t* test. *P* < 0.05 was considered statistically significant.

## Abbreviations

BID: Twice daily; EGFR: Epidermal growth factor receptor; FBS: Fetal bovine serum; HRP: Horseradish peroxidase; HUVECs: Human umbilical vein endothelial cells; IC_50_: Half maximal inhibitory concentration; NSCLC: Non–small-cell lung cancer; PBS: Phosphate-buffered saline; PDGFR: Platelet derived growth factor receptor; QD: Once daily; QW: Once weekly; RMANOVA: Repeated-measures analysis of variance; VEGF: Vascular endothelial growth factor; VEGFR: Vascular endothelial growth factor receptor.

## Competing interests

AC, BZ, SK, MX, HW, DW, JS, HS, CS, DS, and AP are employees of and shareholders in Amgen Inc.

## Authors’ contributions

AC: Study conception and design, data analysis and interpretation, writing and revising the manuscript. BZ: Xenograft experiments, data analysis, and revising the manuscript. SK: Histomorphometric analysis of xenograft studies, data analysis, and revising the manuscript. MX: Xenograft experiments and revising the manuscript. HW: Xenograft experiments and revising the manuscript. DW: Xenograft experiments and revising the manuscript. JS: In vitro proliferation assays and Western blots; data analysis, and revising the manuscript. HS: Xenograft experiments and revising the manuscript. CS: Study conception and design, data analysis and interpretation, revising the manuscript. DS: Study conception and design, data analysis and interpretation, revising the manuscript. AP: Study conception and design, data analysis and interpretation, writing and revising the manuscript. All authors read and approved the final manuscript.

## Supplementary Material

Additional file 1 **Figure S1.** Effects of treatment with an Amgen proprietary small-molecule VEGF receptor inhibitor (“Compound 72”) on lung mass in a KRAS-driven genetically engineered mouse model of lung adenocarcinoma. In this model, development of lung tumors was induced by intratracheal delivery of adenovirus containing the Cre-recombinase to *KRAS*^*LSL-G12D*^ mice. Animals with established lung tumors were treated with (**A**) vehicle (n = 12) or (**B**) small-molecule VEGF receptor inhibitor 30 mg/kg QD (n = 10). Figure S2 Representative computed tomography images of mice with mutant *KRAS* G12D lung cancer (as described in Additional file 1) 11 weeks after induction of disease and 3 weeks after treatment with an Amgen proprietary small-molecule VEGF receptor inhibitor (“Compound 72”). (**A**) Vehicle. The image shows wide-spread tumor burden and minimal viable lung space. (**B**) Treatment with a small-molecule VEGF receptor inhibitor resulted in visible preservation of normal, viable lung with less tumor burden. Click here for file
