# Supplementary material for: Antitumor activity of motesanib alone and in combination with cisplatin or docetaxel in multiple human non–small-cell lung cancer xenograft models
Source: Mol Cancer. 2012 Sep 19;11:70. doi: 10.1186/1476-4598-11-70 (PMC3515409; doi:10.1186/1476-4598-11-70)

# Antitumor activity of motesanib alone and in combination with cisplatin or docetaxel in multiple human non–small-cell lung cancer xenograft models

Angela Coxon, Beth Ziegler, Stephen Kaufman, Man Xu, Hongyu Wang, Dawn Weishuhn, Joanna Schmidt, Heather Sweet, Charlie Starnes, Douglas Saffran, Anthony Polverino

# Supplemental Material

**Supplemental Figure 1** Effects of treatment with an Amgen proprietary small-molecule VEGF receptor inhibitor (“Compound 72”) on lung mass in a *KRAS*-driven genetically engineered mouse model of lung adenocarcinoma. In this model, development of lung tumors was induced by intratracheal delivery of adenovirus containing the Cre-recombinase to *KRAS^LSL-G12D^* mice Animals with established lung tumors were treated with (**A**) vehicle (n = 12) or (**B**) small-molecule VEGF receptor inhibitor 30 mg/kg QD (n = 10).


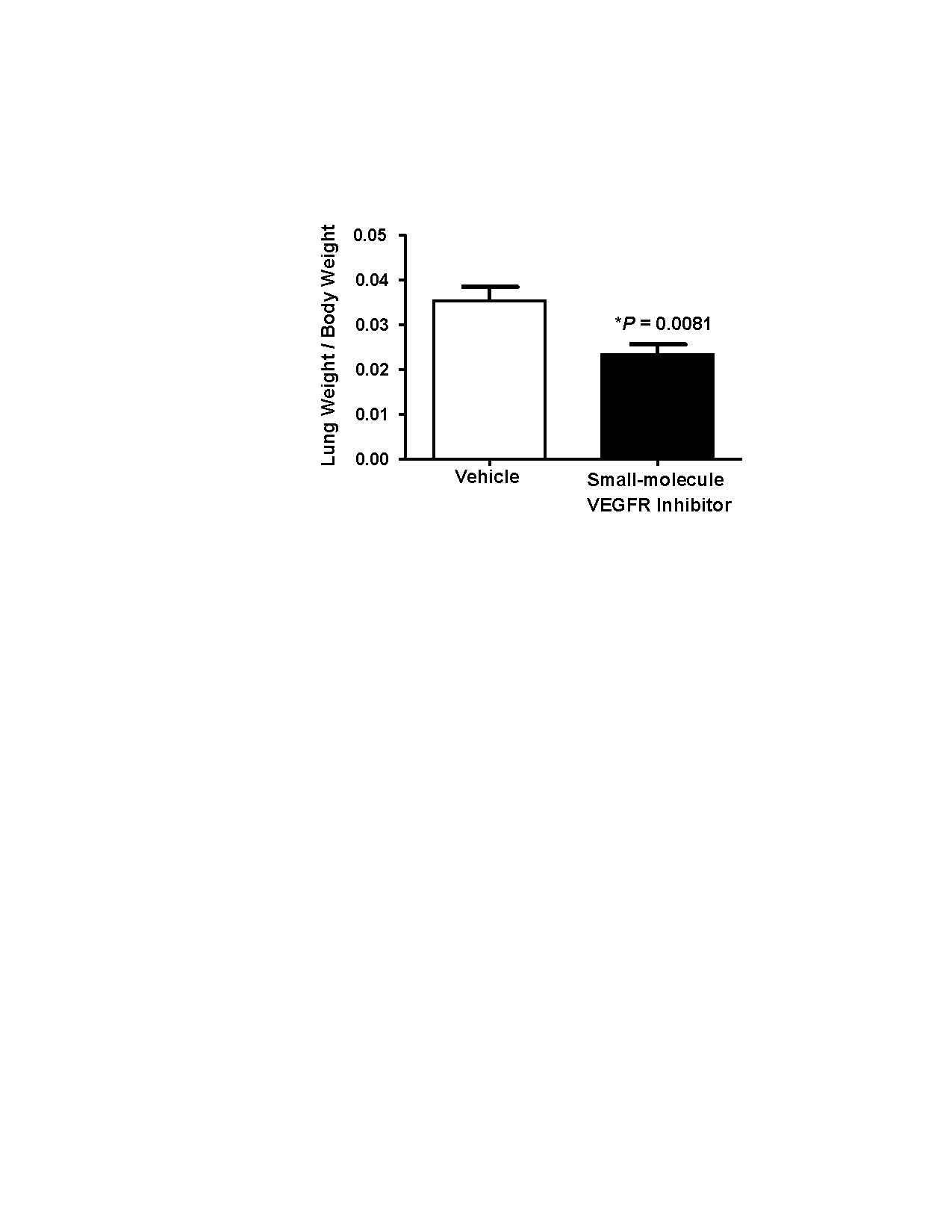


**Supplemental Figure 2** Representative computed tomography images of mice with mutant *KRAS* G12D lung cancer (as described in Supplemental Figure 1) 11 weeks after induction of disease and 3 weeks after treatment with an Amgen proprietary small-molecule VEGF receptor inhibitor (“Compound 72”). (**A**) Vehicle. The image shows wide-spread tumor burden and minimal viable lung space. (**B**) Treatment with a small-molecule VEGF receptor inhibitor resulted in visible preservation of normal, viable lung with less tumor burden.


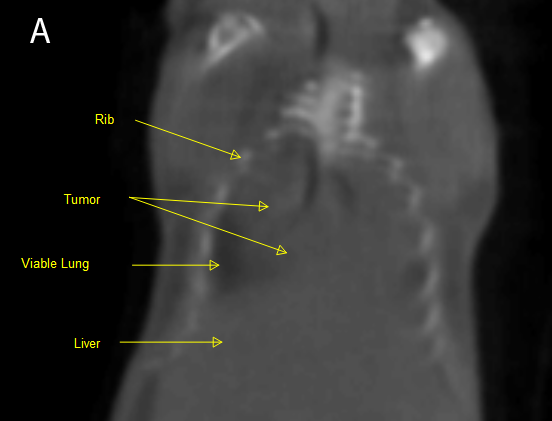

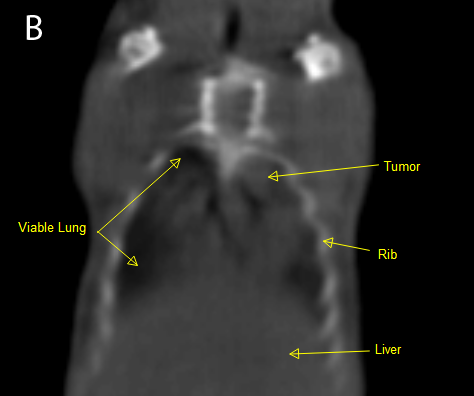

Supplement: Additional file 1 — Figure S1. Effects of treatment with an Amgen proprietary small-molecule VEGF receptor inhibitor (“Compound 72”) on lung mass in a KRAS-driven genetically engineered mouse model of lung adenocarcinoma. In this model, development of lung tumors was induced by intratracheal delivery of adenovirus containing the Cre-recombinase to KRASLSL-G12D mice. Animals with established lung tumors were treated with (A) vehicle (n = 12) or (B) small-molecule VEGF receptor inhibitor 30 mg/kg QD (n = 10). Figure S2 Representative computed tomography images of mice with mutant KRAS G12D lung cancer (as described in Additional file 1) 11 weeks after induction of disease and 3 weeks after treatment with an Amgen proprietary small-molecule VEGF receptor inhibitor (“Compound 72”). (A) Vehicle. The image shows wide-spread tumor burden and minimal viable lung space. (B) Treatment with a small-molecule VEGF receptor inhibitor resulted in visible preservation of normal, viable lung with less tumor burden. [file 1476-4598-11-70-S1.doc]
